# Effect of Pt on Stress Rupture Properties of Pt-Modified Nickel Aluminide Coatings at 1100 °C

**DOI:** 10.3390/ma17071520

**Published:** 2024-03-27

**Authors:** Youying Xue, Bin Yin, Peng Deng, Chunming Deng, Jie Mao, Zhaoguo Qiu, Dechang Zeng, Min Liu

**Affiliations:** 1 School of Materials Science and Engineering, South China University of Technology, Guangzhou 510640, China; xueyouying@163.com (Y.X.); medczeng@scut.edu.cn (D.Z.); 2 The Key Lab of Guangdong for Modern Surface Engineering Technology, National Engineering Laboratory for Modern Materials Surface Engineering Technology, Institute of New Materials, Guangdong Academy of Sciences, Guangzhou 510651, China; dp02163@163.com (P.D.); dengchunming@gdinm.com (C.D.); maojie@gdinm.com (J.M.); liumin@gdinm.com (M.L.); 3 State Key Laboratory of Powder Metallurgy, Central South University, Changsha 410083, China

**Keywords:** Pt-modified nickel aluminide coatings, superalloy, Pt content, stress rupture life, elevated temperatures

## Abstract

Platinum plays a crucial role in the superior high-temperature oxidation resistance of Pt-modified nickel aluminide (PtAl) coatings. However, PtAl coatings usually serve in thermo-mechanical coupling environments. To investigate whether Pt contributes to the high-temperature mechanical properties of PtAl coating, stress rupture tests under 1100 °C/100 MPa were performed on PtAl coatings with varying Pt contents. The different coatings were obtained by changing the thickness of the electroplated Pt layer, followed by a diffusion heat treatment and the aluminizing process in the present work. The results of the stress rupture tests indicated that an increasing Pt content resulted in a significant decrease in the stress rupture life of PtAl-coated superalloys under 1100 °C/100 MPa. Theoretical calculations and microstructural analysis suggested that an increased coating thickness due to the Pt content is not the main reason for this decline. It was found that the cracks generated close to the substrate in high-Pt-coated superalloys accelerated the fracture failure.

## 1. Introduction

Pt-modified nickel aluminide (PtAl) coatings are widely applied to nickel-based superalloy components operating in hot sections of advanced gas turbine engines, owing to their excellent high-temperature oxidation resistance [1]. The superior oxidation resistance is mainly derived from the role of Pt in the coating, which promotes the formation and healing of protective alumina scales [2,3,4,5,6,7]. However, investigating the durability of PtAl coatings in complex service environments, such as the interactions of temperature, stress, and chemical field, based solely on the oxidation resistance of the coatings is still not sufficient.

Currently, researchers have also conducted some studies on the failure behavior of PtAl coatings under complex muti-field coupling environments [8,9,10,11,12,13,14,15,16,17]. Most of them concentrated on the impact of different loading conditions, such as tension [8,9,10,11], creep [9,10,11,12,13,14,15], fatigue [16,17,18], and thermo-mechanical fatigue [19], on the mechanical properties of PtAl coatings from room temperature to the service temperature. In general, except for high-cycle fatigue at a high temperature, depositing the PtAl coating would decrease the mechanical strength of the substrate superalloy but improve its ductility at elevated temperatures.

However, the limited literature revealed the effect of the original composition on the stress rupture properties of PtAl coatings. Taylor et al. [20] found that the creep strength of a PtAl coating was significantly higher than that of similar coatings. They owed the better mechanical performance to the discrepancy of the original platinum and aluminum content between the coatings but without a deeper explanation. Alam et al. [21] found that the tensile strength of a free-standing PtAl coating increased with the increasing Pt from room temperature to 1100 °C. However, according to the research conducted by Barjesteh et al. [22], an increased Pt content in the PtAl coating led to a significant reduction in the tensile strength of the coated superalloys at 871 °C. This was due to the higher tensile residual stress induced by a more severe thermal mismatch between the substrate and the coating in the high-Pt coating.

In this paper, the effect of Pt on the stress rupture properties of the PtAl-coated superalloys under 1100 °C/100 MPa was investigated. An unexpected result was found that a coating with a high Pt content had worse stress rupture properties despite being thought to perform well in resisting oxidation [2,3,4,5,6,7]. To further understand the relationship between the composition of PtAl coatings and the high-temperature service performance of coated superalloys, a detailed study was carried out. 

## 2. Materials and Methods

### 2.1. Preparation of Different Pt-Content PtAl Coatings

A commercial third-generation Ni-based single-crystal superalloy was used in the present work. Its normal composition (in wt.%) is 3.5Cr, 7Co, 15.5 (Mo + W + Ta), 4.5Re, 0.5Nb, 6Al, a trace amount of Hf, C, Y, and balanced Ni. To better simulate the shape of the turbine blade, the specimens were cut into a sheet shape, as reported [23], by electrical discharge machining along the [001] direction of the single crystal rods, with the dimensions of a gauge length of 10 mm, 2 mm in width, and 1 mm in thickness. 

The specimens were firstly ground from 180# to 1000# SiC papers, and then wet-blasted with alumina grit. To obtain PtAl coatings with different Pt contents, Pt layers of three different thicknesses [24], namely 0, 3, and 7 μm, were electrodeposited on the sheets. The electroplating of the Pt layer used the alkaline Pt(NH_3_)_2_(NO_2_)_2_ plating solution with a concentration of 12 g/L at around 90 °C, and the current density was controlled at 1–2 A/dm^2^. The thickness of the plated layer was calculated via weighing. The plated sheet was then subjected to a diffusion heat treatment in a vacuum ($p_{O_{2}}$ < 10^−5^ mbar) at 1050 °C for 4 h. Afterward, an “above pack” aluminization treatment was conducted using a mixture of 98 wt.% commercial Fe-50 wt.% Al powder and 2 wt.% NH_4_Cl at 1050 °C for 5 h in an argon atmosphere. Henceforth, the coatings corresponding to the initial Pt layer thicknesses of 0, 3, and 7 μm are referred to as 0 Pt, 3 Pt, and 7 Pt coating, respectively. 

### 2.2. Stress Rupture Tests and Characterizations

The stress rupture tests were carried out in air under 1100 °C/100 MPa for both coated and uncoated specimens. The heating rate of the tests was about 10 °C/min. To ensure the temperature field was uniform, the specimens were kept at 1100 °C for an hour before loading. 

The phase constitution of the coatings was examined by X-ray diffraction (Smart Lab, Rigaku, Tokyo, Japan) with a scanning range of 10–90° (4°/min). The cross-sectional morphology of the as-fabricated and fracture specimens was obtained with a scanning electron microscope (FE-SEM, NanoSEM 450, Hillsboro, OR, USA). The composition of each zone of the coatings was analyzed by energy-dispersive spectroscopy (AMETEK EDAX, Warrendale, PA, USA), and no less than three areas were selected to obtain the average composition. 

The Schmid factor distribution maps were statistically obtained by the SEM equipped with an electron back-scattered diffraction machine (Gemini SEM 300 with the Symmetry S2 detector, Oxford, UK) using Channel 5 software. Before the EBSD studies, the specimens were ground and vibration-polished in a SiO_2_ polishing solution for 3 h to reduce the residual stress. The patterns were captured with a scanning step of 0.1 μm. 

The hardness (HV) and Young’s modulus (E) of every area of the specimens were obtained by the nano-indentation (NHT3, Anton Paar, Graz, Austria). Before the tests, the specimens underwent strict grinding and polishing to ensure a smooth surface and minimal residual stress. The pressure head applies a load to the processed specimens at a constant loading rate of 20 mN/min. After reaching the maximum load of 10 mN, the load is maintained for 10 s and then unloaded. At least 5 indentation points were collected in each area to obtain the average hardness and Young’s modulus.

## 3. Results and Discussion

**Figure 1:**
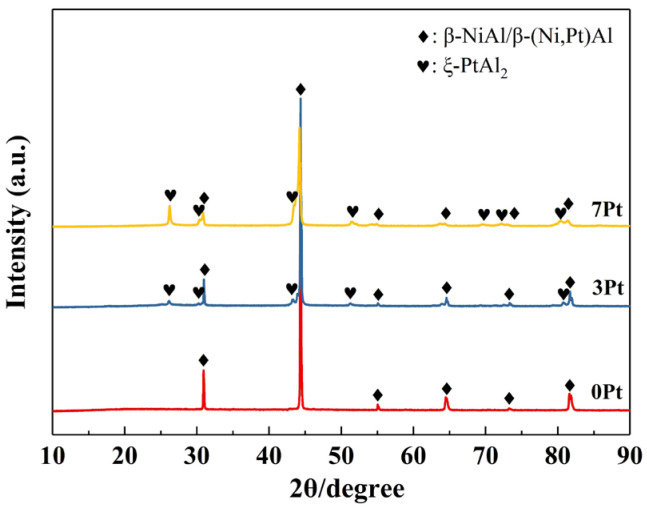
The XRD patterns of the as-fabricated PtAl coatings with different Pt contents.

Figure 1 exhibits the phase constituents of the as-fabricated PtAl coatings with different Pt contents. The 0 Pt coating is mainly composed of the β-NiAl phase. With the addition of Pt, the β-(Ni, Pt)Al phase forms in the coatings due to the high solubility of Pt in β-NiAl (a solubility limit of ~42 at.%) [25]. In addition, a small content of ξ-PtAl_2_ phase is also present in the coatings.

**Figure 2:**
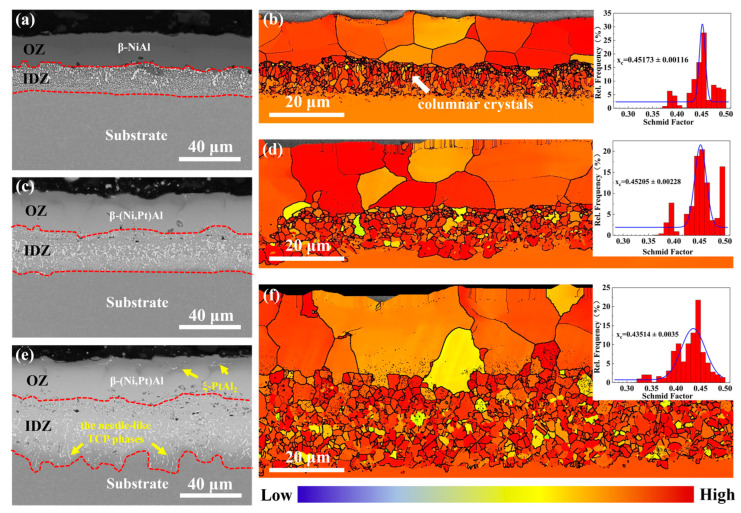
Cross-sectional microstructures of the as-fabricated (**a**) 0 Pt, (**c**) 3 Pt, and (**e**) 7 Pt coatings. The corresponding grain boundary maps and Schmid factor distribution maps of the {110} <111> slip systems are shown in (**b**,**d**,**f**). The upper right picture is the statistical Schmid factor distribution of the OZ in the coatings.

Figure 2 displays the cross-sectional microstructure of the as-fabricated PtAl coatings with different Pt contents (a, c, and e). All the coatings exhibit the typical double-layer structure. The outer zone (OZ) of the coatings mainly consists of the β-NiAl or β-(Ni, Pt)Al phase, as depicted in Figure 1. The interdiffusion zone (IDZ) in the coatings is filled with the topologically closed phases (TCP) that are rich in Ta, W, Re, and other refractory elements, as shown in Table 1. As the Pt level of the coatings continuously increases, a trace number of ξ-PtAl_2_ phases appear on the surface, and numerous needle-like TCP phases form in the IDZ. Moreover, the TCP phases become larger and more dispersed, along with the increasing Pt content. From the grain boundary map of 0 Pt coating (Figure 2b), many fine columnar crystals appear at the interface between the IDZ and OZ. With the increase in Pt content, the columnar grains gradually grow and change into coarse equiaxed crystals (Figure 2d). The Schmid factor distribution map is commonly used to characterize the ease of activation of slip systems in specimens under stress [16]. As shown in Figure 2b,d,f, the OZ of the 7 Pt coating has the smallest average Schmid factor value (0.435) of the {110} <111> slip systems compared to that of the other two coatings (0.452).

With an increasing Pt content, more Pt can be found simultaneously in the OZ and IDZ of the coatings, as shown in Table 1. In addition, the content of the refractory elements, such as Re and W, diffusing from the substrate to the coating decreases. It indicates that the Pt may effectively inhibit the diffusion of the refractory elements, such as W and Re, from the substrate to the coating, as reported [26]. 

**Figure 3:**
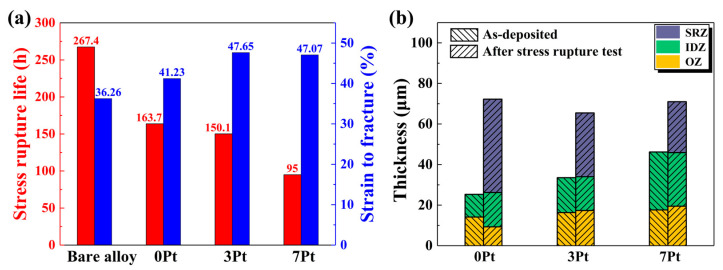
(**a**) The stress rupture life and the fracture strain of the specimens; (**b**) the thickness of each zone of the as-deposited and fracture specimens.

Figure 3a exhibits the stress rupture life and the fracture strain of the specimens under 1100 °C/100 MPa. The stress rupture life declines significantly after the deposition of the PtAl coating, and a further decrease happens with an increasing Pt content of coatings. The fracture strain of the coated specimens increases with the Pt content. Figure 3b shows the thickness of the different regions in the as-deposited and fracture specimens. With an increasing Pt content, the as-fabricated coatings, especially as the IDZ, become thicker. After the stress rupture test, the 0 Pt coating has the thickest secondary reaction zone (SRZ) due to the longest time exposed to the thermo-mechanical coupling environment, while the 7 Pt coating has the thickest IDZ, though with the shortest stress rupture life.

**Figure 4:**
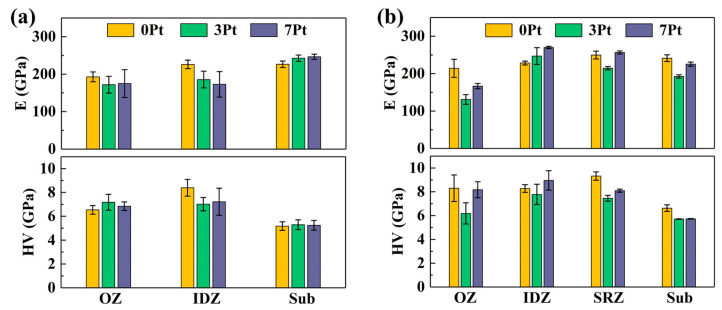
Young’s modulus (E) and hardness (HV) of each zone in the specimens: (**a**) as-fabricated and (**b**) after the stress rupture test under 1100 °C/100 MPa.

Figure 4a displays the mechanical properties of the different regions of the three as-fabricated coated specimens. The hardness of the OZ increases with Pt, and a decline appears in the IDZ of the Pt-contained coating. However, Young’s modulus exhibits a disparate variation trend compared to the hardness. The values of Young’s modulus of the OZ and IDZ decline with increasing Pt, which may relate to the gradually increasing concentration of the Pt_Ni_ and Ni_Al_ antisite defects as reported [27]. Moreover, as shown in Figure 4b, the hardness and Young’s modulus of the OZ in the 3 Pt coating significantly decline after the stress rupture test, while these values exhibit an appreciable increase in the IDZ of the 7 Pt coating.

**Figure 5:**
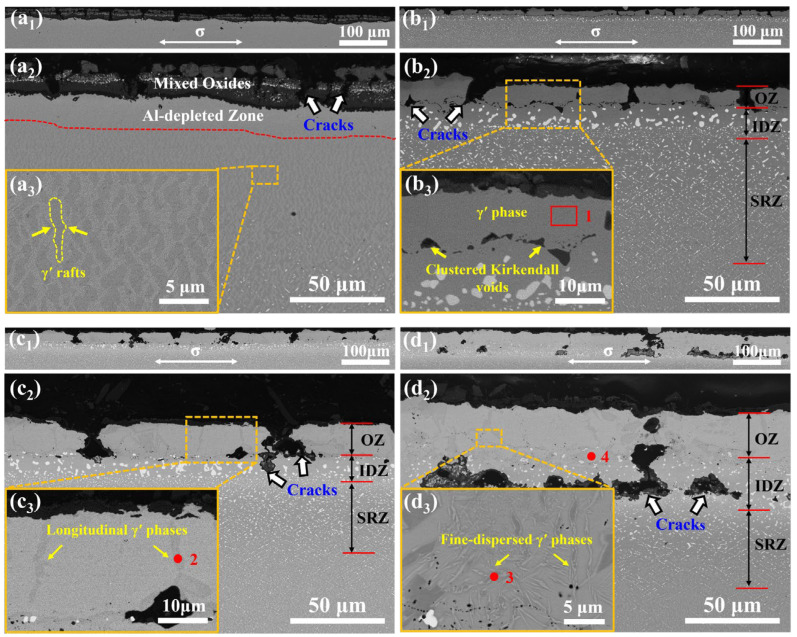
Cross-sectional microstructures of the specimens after the stress rupture test under 1100 °C/100 MPa: (**a_1_**–**a_3_**) the bare alloy, (**b_1_**–**b_3_**) 0 Pt, (**c_1_**–**c_3_**) 3 Pt, and (**d_1_**–**d_3_**) 7 Pt coatings.

Figure 5 presents the cross-sectional morphology of the four specimens that are 2 mm away from the fracture end after the stress rupture test. The compositions of the marked areas are shown in Table 2. As presented in Figure 5a_1–2_, multi-layered mixed oxides form on the surface of the alloy, and an Al-depleted zone is found beneath it, where the γ/γ′ coherent structure disappeared. Moreover, many γ′ rafts oriented perpendicular to the direction of the loading stress appear close to the Al-depleted zone (Figure 5a_3_), which is commonly in the literature [12,13]. As exhibited in Figure 5b_1–2_, the cracks of the 0 Pt coating generate from the surface micro defects, propagate perpendicular to the direction of the stress, and eventually abort at the OZ/IDZ interface, where numerous microvoids exist (Figure 5b_3_). The complete transformation of the β phase into the γ′ phase in the OZ is observed, supported by the composition of Area 1 in Table 2 (21–28 at.% Al in the γ′ phase) [25]. In the 3 Pt coating depicted in Figure 5c_1–2_, the cracks, as well as the number of microvoids in the coating, decrease, but some cracks expand into the IDZ. It can be seen that the γ′ phases (spot 2) penetrate the OZ of the 3 Pt coating and are parallel to the longitudinal cracks (Figure 5c_3_). As shown in Figure 5d_1–2_, the cracks in the 7 Pt coating present a different characteristic compared to the others. Most of them form in the IDZ, and some expand parallel to the direction of the stress. The microvoids at the interface can hardly be seen. In addition, numerous finer γ′ phases (spot 3) are found evenly distributed in the OZ (Figure 5d_3_). From Table 2, the IDZ of the 7 Pt coating maintains a high concentration of Pt content after the stress rupture test compared to the as-fabricated coating (as shown in Table 1).

It was reported that Pt plays a major role in the superior high-temperature oxidation resistance of the PtAl coatings [2,3,4,5,6,7], but an increasing Pt content results in a significant stress rupture life decline of the PtAl-coated superalloys (Figure 3a) in this paper. The abnormal variation in the stress rupture properties among the specimens may result from the increasing coating thickness or the different ways of crack formation and propagation. 

**Figure 6:**
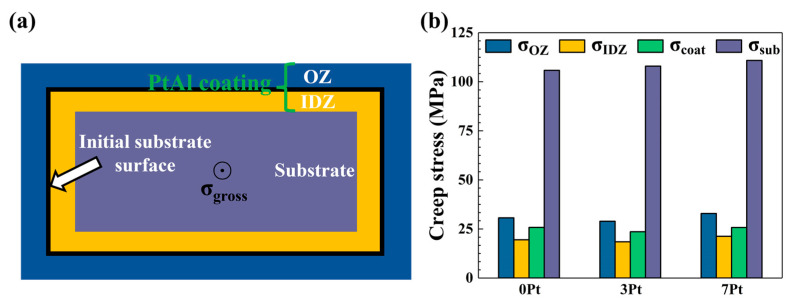
(**a**) The cross-sectional schematic of the as-deposited PtAl-coated superalloy, and (**b**) the calculated creep stress of each zone of the PtAl-coated specimens.

Researchers often obtain the stress distribution of the coated specimen by deconvoluting its overall strain and time response under constant stress [13,20]. To study the effect of the coating thickness on the stress rupture properties of the PtAl-coated superalloy, it is worthwhile to estimate the impact on the stress rupture life of the substrate superalloy after depositing different PtAl coatings by the creep stress of each layer in the coatings. Assuming that the coated specimens were subjected to a constant creep stress $\sigma_{\mathrm{gross}}$; for the muti-layer structural sheet specimens shown in Figure 6a, the creep stress on each part of the coated superalloy has the following relationship with the total stress:

(1) $$\sigma_{\mathrm{gross}}\left(S_{\mathrm{OZ}}+S_{\mathrm{IDZ}}+S_{\mathrm{sub}}\right)=\sigma_{\mathrm{OZ}}S_{\mathrm{OZ}}+\sigma_{\mathrm{IDZ}}S_{\mathrm{IDZ}}+\sigma_{\mathrm{sub}}S_{\mathrm{sub}}$$

For the PtAl coating, there exists:

(2) $$\sigma_{\mathrm{coat}}=\frac{\sigma_{\mathrm{gross}}\left(S_{\mathrm{OZ}}+S_{\mathrm{IDZ}}+S_{\mathrm{sub}}\right)-\sigma_{\mathrm{sub}}S_{\mathrm{sub}}}{S_{\mathrm{OZ}}+S_{\mathrm{IDZ}}}\;$$

Therefore, this formula holds:

(3) $$\sigma_{\mathrm{coat}}\left(S_{\mathrm{OZ}}+S_{\mathrm{IDZ}}\right)=\sigma_{\mathrm{OZ}}S_{\mathrm{OZ}}+\sigma_{\mathrm{IDZ}}S_{\mathrm{IDZ}}$$

The creep stress of the IDZ in the coatings can be expressed as [20]:

(4) $$\sigma_{\mathrm{IDZ}}=\left(1-X\right)\sigma_{\beta }+X\sigma_{\mathrm{tcp}}$$

where the $\sigma_{\mathrm{OZ}}$, $\sigma_{\mathrm{IDZ}}$, $\sigma_{\mathrm{coat}}$, and $\sigma_{\mathrm{sub}}$ represent the creep stress of the OZ, IDZ, the whole coating, and the substrate, respectively. $S_{\mathrm{OZ}}$, $S_{\mathrm{IDZ}}$, and $S_{\mathrm{sub}}$ are the corresponding gauge sectional areas. X is taken to be 0.4 in all cases, according to the previous research [13].

The creep rate of the β and TCP phases in the IDZ can be expressed as [20]:

(5) $$\dot{\varepsilon }=6.3\times 10^{13}(\frac{\sigma_{\beta }}{E}{)}^{4}\exp\;(-\frac{125,000}{\mathrm{RT}})\;$$

(6) $$\dot{\varepsilon }=10.25\;\sigma_{\mathrm{tcp}}^{2}\;\exp\;(-\frac{211,000}{\mathrm{RT}})$$

where the $\sigma_{\beta }$ and $\sigma_{\mathrm{tcp}}$ represent the creep stress of the β and TCP phases in the IDZ; E is Young’s modulus, which can be obtained from Figure 4a; R is the ideal gas constant; and T is the testing temperature. The creep rate $\dot{\varepsilon }$ can be calculated from Figure 3a. 

At an extremely low creep rate, the deformation of each zone of the coating can be regarded as a continuous cooperative process, so the value of $\sigma_{\mathrm{OZ}}$ is considered equal to $\sigma_{\beta }$. The creep stress of each zone in the coated specimens can be obtained by substituting the calculated values into Equations (1)–(4). The results are shown in Figure 6b. It can be seen that the creep stress of the substrate ($\sigma_{\mathrm{sub}}$) among the coated specimens with different Pt contents was not much different. It indicates that the increase in coating thickness due to the increasing Pt content is not the main reason for the significant decrease in the stress rupture life of the coated specimens.

In addition, the PtAl coatings obtained by the high-temperature and low-activity aluminizing process belong to the outward-grown coatings, and the OZ/IDZ interface is roughly the initial substrate surface. It can be seen from Figure 3b that since the thickness of the OZ among the various coatings has not much difference, the total thickness of the specimens is almost the same after the deposition of the coatings. Moreover, the total thickness of the 0 Pt and 7 Pt coatings was not much different after the stress rupture test, although an SRZ formed close to the substrate. Therefore, the change in coating thickness caused by the Pt content was not the main reason for the significant decline in the stress rupture properties of the specimens.

As shown in Figure 5a_2_, the cracks in the uncoated superalloy mainly appear at the micro defects of the mixed oxides and abort at the Al-depleted zone. After the deposition of the coatings, the oxidation resistance of the substrate is enhanced. As a result of the strength of the coating being far inferior to the matrix superalloy [22], cracks are more likely to form under different fracture stresses. In addition, due to the thickness debit effect [28], the creep response of the sheet specimens is sensitive to the changes in the microstructure during the coating preparation process. As a result, the stress rupture life of the coated superalloys significantly declines. 

As exhibited in Figure 5b_2_, the cracks in the 0 Pt coating mainly form at the surface defects and abort at the OZ/IDZ interface. The microvoids at the interface are generated by the process of unequal mass transfer between the coating and substrate, i.e., the Kirkendall mechanism [29]. The Kirkendall voids coalesce into larger holes under the thermo-mechanical coupling (Figure 5b_3_). Stress relaxation occurs at the crack tips when the cracks propagate to the interface. Additionally, the fine columnar crystals near the interface (Figure 2b) play a good role in hindering the further propagation of the cracks [16,30]. Consequently, the cracks abort at the OZ/IDZ interface.

With the addition of platinum, the substitution of Pt for Ni in the β phase leads to an obvious lattice distortion since the atomic radius of Pt is 12% higher than that of Ni [21]. This enhances the coating against plastic deformation, proved by the increased hardness (Figure 4). However, the γ′ phases penetrate the OZ of the 3 Pt coating (Figure 5c_3_) since the grain boundaries are the starting points of the phase transformation. The longitudinal phase boundaries may become the path of crack propagation. Previous studies have shown that the addition of platinum can decrease the diffusion activation energy of aluminum in the β-(Ni, Pt)Al phase and reduce the occurrence of Kirkendall voids [4]. This would inhibit the effect of the clustered Kirkendall voids at the interface to release stress at the crack tips. Moreover, the Pt inhibits the diffusion of the refractory elements from the substrate to the coating, decreasing the number of TCP phases [31], which act as the heterogeneous cores of nucleation. The grains at the OZ/IDZ interface grow up gradually into coarse equiaxed crystals, decreasing the creep resistance of the IDZ [28]. As a result, some of the cracks in the 3 Pt coating expand into the IDZ.

As the Pt content further increases, the Schmid factor of the {111} <110> slip system of the OZ in the 7 Pt coating decreases (Figure 2f), which means the slip system is much harder to start under tensile stress. In addition, the coarse β grains in the OZ provide fewer grain boundaries for the formation of the γ′ phases. As the Al elements continue to be consumed, the fine-dispersed γ′ phases grow within the β grains (Figure 5d_3_) [32], which may hinder the slide or climb of the dislocation, resulting in fewer cracks generated in the OZ of the 7 Pt coating. Additionally, the IDZ in the 7 Pt coating maintains a high concentration of Pt, as shown in Table 2, which would create a large, distorted stress field and interact with the original brittle needle-like TCP phase (Figure 2e) under stress. It is prone to generate stress concentration [33], supported by the appreciatively increased Young’s modulus and hardness after the stress rupture tests (Figure 4). Consequently, the cracks in the 7 Pt coating prefer to be generated in the IDZ and then spread to both ends. The coated superalloy with deeper cracks is thought to have worse mechanical properties due to a great reduction in the effective load-bearing cross-sectional area of the specimens [22]. With an increase in the Pt content in the coating, the locations of the crack initiation and propagation are closer to the substrate. Therefore, the stress rupture life of PtAl-coated superalloys under 1100 °C/100 MPa significantly declined. 

## 4. Conclusions

In summary, a detailed study was conducted on the influence of Pt on the stress rupture properties of a PtAl coating under 1100 °C/100 MPa. The following conclusions can be drawn:

1. The increase in Pt content leads to a continuous decrease in the stress rupture life of the PtAl-coated superalloy. Compared to the 0 Pt coating, the stress rupture life decreases by nearly 42% in the 7 Pt coating, while the fracture strain increases from 41.23% to 47.07%.
2. Clustered Kirkendall voids and fine columnar crystals at the OZ/IDZ interface of 0 Pt coating can hinder crack propagation.
3. The interface of the longitudinal γ′ phase in 3 Pt coating can easily become the path for crack propagation.
4. The needle-like TCP phases in the IDZ of 7 Pt coating cause severe stress concentration during creep, promoting the formation of cracks near the substrate and accelerating fracture failure.

## Figures and Tables

**Table 1:** The average composition of each zone in the as-fabricated coatings (at.%).

Specimen	Area	Al	Mo	Cr	Co	Ni	Ta	W	Re	Pt
0 Pt	OZ	36.11	0	0.96	4.05	58.87	0	0	0	0
	IDZ	25.87	0.75	5.58	7.24	53.13	2.36	3.21	1.85	0
3 Pt	OZ	38.62	0	1.87	4.02	49.29	0	0	0	6.20
	IDZ	25.48	0.27	5.49	6.46	50.76	2.92	2.25	1.28	5.10
7 Pt	OZ	39.27	0	2.20	3.74	44.71	0	0	0	10.08
	IDZ	21.45	0.16	4.90	6.40	49.90	3.70	1.94	1.39	10.16

**Table 2:** The composition of the marked areas in Figure 5 that obtained by EDS (at.%).

Area	Al	Mo	Cr	Co	Ni	Ta	W	Pt
1	24.25	0	2.28	5.46	66.80	1.22	0	0
2	23.48	0	1.82	4.14	65.63	0	1.86	4.34
3	24.71	0	1.15	3.37	62.53	0.13	0.14	7.91
4	22.42	0.15	1.67	4.73	60.42	2.95	0.83	6.82

## Data Availability

The data presented in this study are available on request from the corresponding authors.
